# Which support from the French Foundation of rare disease towards clinical trial set up in rare diseases?

**DOI:** 10.1186/1750-1172-10-S2-O33

**Published:** 2015-11-11

**Authors:** Luigi Ravagnan

**Affiliations:** 1 French Foundation for rare diseases, Paris, France

## 

The French Foundation for rare diseases (*Fondation maladies rares*) is a new private non-profit organisation started in 2012 by Pr. Nicolas Lévy and Céline Hubert, which pooled together their complementary experiences in the field of rare diseases, from academia and the pharmaceutical industry respectively. Headquartered in Paris at the heart of the ‘Rare Diseases Platform’, the Foundation reaches out to the whole national territory with its network of regional delegates. The team is now composed of 14 dedicated professionals.

The Foundation was foreseen in the 2^nd^ French National Rare Diseases Plan, as the flagship measure of the research axis. It was created and financially supported by 5 founders representing the patients, the research sector and the medical sector (AFM-Téléthon, Alliance Maladies Rares, National Institute of Health and Medical Research - Inserm, Conference of University Presidents – CPU and Conference of University Hospitals Directors-General).

The Foundation carries out a mission of general interest: it aims at accelerating rare diseases research programs by improving the coordination among rare diseases players, contributing to the understanding of rare diseases, the development of new treatments and the improvement of patient's care and lives.

Since its creation, 168 research projects were funded, for an amount granted in excess of €4M, and over 100 ‘proofs of concepts’ detected, half of which actively followed to help fill the gaps towards clinical development (e.g. strengthening of the proof of concept, orphan drug designation, agreement with a private partner, European funding, etc.).

